# Women's utilisation of quality antenatal care, intrapartum care and postnatal care services in Ethiopia: a population-based study using the demographic and health survey data

**DOI:** 10.1186/s12889-023-15938-8

**Published:** 2023-06-19

**Authors:** Melese Girmaye Negero, David Sibbritt, Angela Dawson

**Affiliations:** 1 School of Public Health, Institute of Health Sciences, Wollega University, Nekemte, Ethiopia; 2 School of Public Health, Faculty of Health, University of Technology Sydney, Sydney, Australia

**Keywords:** Antenatal care, Intrapartum care, Postnatal care, Maternal health, Quality of care, Ethiopia

## Abstract

**Objective:**

This study sought to investigate the level and determinants of receiving quality antenatal care (ANC), intrapartum care, and postnatal care (PNC) services by women in Ethiopia. The quality of care a woman receives during ANC, intrapartum care, and PNC services affects the health of the woman and her child and her likelihood of seeking care in the future.

**Methods:**

Data from the nationally representative Ethiopia Mini Demographic and Health Survey 2019 were analysed for 5,527 mothers who gave birth within five years preceding the survey. We defined quality ANC as having: blood pressure measurement, urine and blood tests, informed of danger signs, iron supplementation, and nutritional counselling during ANC services; quality intrapartum care as having: a health facility birth, skilled birth assistance, and a newborn put to the breast within one hour of birth during intrapartum care services; and quality PNC as having: PNC within two days; cord examination; temperature measurement, and counselling on danger signs and breastfeeding of the newborn; and healthcare provider's observation of breastfeeding during PNC services. We used multilevel mixed-effects logistic regression analyses specifying three-level models: a woman/household, a cluster, and an administrative region to determine predictors of each care quality. The analyses employed sampling weights and were adjusted for sampling design.

**Results:**

Thirty-six percent (*n* = 1,048), 43% (*n* = 1,485), and 21% (*n* = 374) women received quality ANC, intrapartum care and PNC services, respectively. Private healthcare facilities provided higher-quality ANC and PNC but poor-quality intrapartum care, compared to public health facilities. Having four or more ANC visits, commencing ANC during the first trimester, and higher women's education levels and household wealth indices were positive predictors of quality ANC use. Government health posts were less likely to provide quality ANC. Wealthier, urban-residing women with education and four or more ANC contacts were more likely to receive quality intrapartum care. Women who received quality ANC and skilled birth assistance were more likely to receive quality PNC. Teenage mothers were more likely to receive quality intrapartum care, but were less likely to receive quality PNC than mothers aged 20–49.

**Conclusions:**

We recommend standardizing the contents of ANC provided in all healthcare facilities; and promoting early and four or more ANC contacts, effectiveness, sensitivity and vigilance of care provided to teenage mothers, and women's education and economic empowerment.

## Background

Reducing the unacceptably high maternal and perinatal morbidity and mortality rates in low-income countries requires considerable investment to increase access to, demand for, and use of skilled maternity care, alongside enhancing the quality of care delivered [1]. In Ethiopia in 2017, 49% of 1010 reported maternal deaths occurred after women arrived at healthcare facilities. Fourteen percent of these maternal deaths were attributed to a lack of supplies and equipment, 11% to delays in patient management at the facility, 6% to healthcare provider error and mismanagement, and 28% to referral delays from other facilities [2]. Therefore, increasing access to, and utilization of, maternal healthcare alone is insufficient to improve maternal health outcomes [3]. The quality of care a woman receives across ANC, intrapartum care, and PNC services affects the health of the woman and her child and her likelihood of seeking care in the future [3, 4]. Measuring the quality of existing maternal healthcare and identifying its determinants are essential for planning improvements in current and future care [3].

While there has been a strong focus on improving access to healthcare during pregnancy, labour and delivery, and postnatal periods, there has been less emphasis on ensuring effective coverage or content with the provision of all the recommended interventions during antenatal, intrapartum, and PNC services. This has resulted in missed opportunities to alleviate maternal and newborn morbidities and mortalities [5]. Reaching the 2030 Sustainable Development Goals (SDGs) target of reducing the global maternal mortality ratio to less than 70/100,000 live births and the global neonatal mortality rates to less than 12/1,000 live births in Ethiopia requires a rapid improvement in maternal healthcare quality [6].

Maximizing the life-saving potential of ANC in low-resource settings requires a focus on quality. For many women around the globe, an ANC visit may be their first adult contact with the healthcare system. ANC, therefore, serves as a gateway to health services both during and beyond maternity care. In addition to diagnosing and managing pregnancy-related complications, ANC provides an opportunity to screen for and treat other chronic conditions and non-communicable diseases [7]. However, in low-income settings, the mere focus on the percentage of mothers receiving four or more ANC contacts as a global benchmark indicator to track maternal health program performance than on the content and process of ANC is limiting the ability to early identify and address complications and maximize health outcomes [8].

Over the last two decades, women have been encouraged to give birth in healthcare facilities to ensure access to skilled personnel and timely referral if required. However, giving birth in a healthcare facility may not guarantee quality care [9]. Disrespectful care has been reported in facilities that not only violate a woman's human rights but are a significant barrier to accessing future intrapartum care services [9, 10]. A negative experience in childbirth is associated with post-traumatic stress disorder, disruption to interpersonal relationships, and dysfunctional maternal-infancy bonding [11, 12].

Women and their newborns require support and careful monitoring after birth. Most maternal and infant deaths occur in the first six weeks after delivery, yet this remains a neglected area of care [13]. Basic care for all newborns should include promoting and supporting early and exclusive breastfeeding if possible, keeping the baby warm, increasing handwashing, and providing hygienic umbilical cord and skincare. Families should be counselled to identify danger signs, understand the care that both the woman and newborn need, and know where to reach services when needed. Promoting a healthy lifestyle and good nutrition, detecting and preventing diseases, supporting women who may be experiencing intimate partner violence, and ensuring access to sexual and reproductive health, including postpartum family planning, are also key to quality postnatal care [13].

Quality prenatal, intrapartum, and postnatal care are vital maternal healthcare services that should be delivered by skilled personnel. Quality ANC, according to the World Health Organization (WHO), includes nutritional counselling and multivitamin supplements, frequent visits (eight or above ANC contacts), blood and urine tests, preventive antibiotics, tetanus toxoid injections, and health education on pregnancy and birth danger signs [14, 15]. Respectful care, clear and compelling communication between the woman and her healthcare provider, the option of a companion during labour and delivery, delivery at a healthcare facility, skilled personnel assistance, appropriate pain relief strategies, mobility in labour where possible, choice of birth position, use of uterotonics, delayed cord clamping (after a minute), immediate kangaroo care and breastfeeding, delayed bathing of the newborn (24 h), and the care of mother and newborn in a healthcare facility for at least 24 hours after delivery are all components of quality intrapartum care [15, 16]. Immediate PNC within 24 h of birth and at least three additional PNC visits for the mother and the newborn within 42 days of birth, home visits in the first week after birth, exclusive breastfeeding, cord care, prophylactic antibiotics for the mother, and health education on maternal and newborn health danger signs are all components of quality PNC [15, 17].

Measuring the existing maternal healthcare quality and its determinants at a country level, using the nationally representative demographic and health survey (DHS) data, can identify gaps in care and provide insight into reducing maternal and newborn morbidity and mortality [18, 19]. There is currently no research that provides a comprehensive and standard view of quality maternal healthcare services across ANC, intrapartum care and PNC services in Ethiopia to inform health service planning and improve health outcomes.

One study focused on the quality of ANC and PNC in 20 sub-Saharan African countries, including Ethiopia. This research, based on a secondary analysis of DHSs data, revealed that while 51% of mothers received four or more ANC visits with at least one visit from skilled personnel, only 5% received eight ANC interventions (blood pressure measurement, urine and blood test, iron supplementation, tetanus protection, counselling on pregnancy complications, HIV testing and results, and three doses of intermittent preventive treatment of malaria in pregnancy). While 65% of births in this study were attended by skilled personnel, no data is provided concerning the interventions provided during intrapartum care. Only 3% of women received all seven PNC interventions (newborn weighed at birth, early initiation of breastfeeding, no pre-lacteal feed, BCG and polio vaccines, and PNC for mother and newborn within two days of birth) [5].

While no research has examined the quality of care across the three packages of maternal healthcare, two studies by Bayou et al. (2016) and Gebrekirstos et al. (2021) have assessed adequate ANC among slum residents in Addis Ababa and Southern Ethiopia. These authors defined quality ANC as commencing ANC during the first trimester, four or more ANC contacts, weight, height and blood pressure measurements, urine and blood tests, tetanus injection, iron supplementation, and counselling on pregnancy complications. In this study, only 11% and 23% had adequate ANC in Addis Ababa and Southern Ethiopia, respectively [20, 21]. Based on the then most recent (2007–16) DHSs and Multiple Indicator Cluster Surveys data in 91 low and middle-income countries, Arsenault et al. (2018) described quality ANC according to the receipt of three essential services (blood pressure measurement, urine examination, and blood testing) among women who had at least one visit with a skilled ANC provider [22].

In addition, one study investigated the effect of user-friendly mobile health (mHealth) solution technology in improving the timeliness and quality of maternal and newborn healthcare services in the primary healthcare system in Ethiopia by bridging communication gaps between other healthcare workers and the health extension workers. Accordingly, the routine assessment showed that the mHealth application facilitated real-time information exchange with supervising healthcare facilities in the primary healthcare system and timely identification and registration of pregnant women, thereby increasing the uptake of critical maternal healthcare interventions across the continuum from pregnancy to childbirth and postpartum [23]. Furthermore, a secondary government data analysis study on the role of a computerized clinical decision support system in improving the quality of intrapartum and postnatal care services in India showed a significant improvement in adherence to key clinical practices, reduction in incidence still births rates and asphyxia, increase in identification of maternal complications and decrement in referral outs after the introduction of the technology [24]. Our study aimed to assess the quality of antenatal care (ANC), intrapartum care, and postnatal care (PNC) services and to identify their multifaceted determinants in Ethiopia using data from the 2019 Ethiopia Mini DHS (MDHS). We therefore comprehensively assessed the recommended interventions during ANC, intrapartum care, and PNC services a mother and/or her newborn received and the associated socio-demographic determinants at a national level in Ethiopia using the 2019 Ethiopia MDHS data.

## Methods

### Study setting

Ethiopia, the oldest independent African nation with rich geographical diversity that includes rugged mountains, flat-topped plateaus, deep gorges, and river valleys, is located in the centre of the Horn of Africa. More than half of the country's geographic area lies 1,500 meters above the sea level [25]. It is bordered by Sudan and South Sudan on the west, Eritrea and Djibouti on the northeast, Somalia on the East and Southeast, and Kenya on the south. The country covers an area of 1,126,829 square kilometres. It is a Federal Democratic Republic composed of nine National Regional states: namely Tigray, Afar, Amhara, Oromia, Somali, Benishangul-Gumuz, Southern Nations Nationalities, and Peoples' Region (SNNPR), Gambella and Harari, and two Administrative states Addis Ababa City administration and Dire Dawa city council. In Ethiopia, the sex ratio between males and females is almost equal (100.87), and women of reproductive age constitute about 23% of the total population [25]. Ethiopia is a low-income country with a gross domestic product (GDP) per capita (current US$) of $772 in 2018, up from about $340 in 2010. Nearly 80% of the Ethiopian population lives in rural areas, mainly dependent on subsistence agriculture [25]. As of 1 July 2019, Ethiopia had a total population of 112,079,000, with a projection for 2030 of 144,944,000, and is the second most populous country in Africa after Nigeria [26].

### Data sources

We used the 2019 Ethiopia Mini Demographic and Health Survey (EMDHS) data conducted by the Ethiopian Public Health Institute (EPHI) from 21 March 2019 to 28 June 2019, with permission from the DHS program. The DHS is a nationally representative cross-sectional study conducted every five years in over 85 low-income and middle-income countries across the globe since 1984 [27, 28]. The data for all the analyses were extracted from the 2019 Ethiopia MDHS children's recode (KR) files. The main objectives of the 2019 EMDHS were to collect nationally representative high-quality data house to house and provide up-to-date estimates on key demographic and health indicators in Ethiopia: breastfeeding; maternal and child health (ANC, delivery, and PNC); infant, child, and neonatal mortality levels; child nutrition; and other health issues relevant to the achievement of the SDGs [29].

### Sampling and sample size

The 2019 EMDHS used the sampling frame of all census enumeration areas (EAs) created for the 2019 Ethiopia Population and Housing Census (EPHC) and conducted by the Central Statistical Agency (CSA). The census frame was a complete list of 149,093 EAs created for the 2019 EPHC. An EA is a geographic area covering an average of 131 households. The 2019 EMDHS sample provides estimates of key indicators for the country as a whole, for urban and rural areas separately, and for each of the nine regions and the two administrative cities [29].

The DHS employs a multi-stage cluster sampling technique to select women of reproductive age (15–49 years) and children younger than five years for inclusion [27, 28]. The 2019 EMDHS employed a two-stage cluster sampling technique. To ensure that survey precision was comparable across regions, sample allocation was done through an equal allocation wherein 25 EAs were selected from eight regions. However, 35 EAs were selected from each of the three larger regions: Amhara, Oromia, and the Southern Nations, Nationalities, and Peoples' Region (SNNPR) [29]. In the first stage, 305 EAs (93 in urban and 212 in rural areas) were selected with probability proportional to EA size and independent selection in each sampling stratum. A household listing operation was then carried out in all selected EAs from January through April 2019. In the second stage of selection, a fixed number of 30 households per cluster were selected with an equal probability of systematic selection from the newly created household listing. All women aged 15–49 years, either permanent residents of the selected households or visitors who slept in the household the night before the survey, were eligible to be interviewed. In the interviewed households, 9,012 eligible women were identified for individual interviews; interviews were completed with 8,885 women, yielding a response rate of 99% [29]. We used information on weighted samples of 5,527 women surveyed with at least one live birth within five years preceding the 2019 EMDHS for the analyses.

### Outcome variables

Three outcome variables were included in the study: the women's utilization of quality antenatal care, intrapartum care and postnatal care services based on the WHO-recommended interventions during ANC, intrapartum care, and PNC services that are available in the DHS dataset. Accordingly, having blood pressure measurement, urine tests for detecting bacteriuria and proteinuria, blood tests for infection and anaemia, information about the danger signs of pregnancy, provision of iron supplements, and provision of nutritional counselling for women who received ANC services was considered a proxy for having received quality ANC [30–32]. A woman would need to receive all six of these interventions to be considered to have received quality ANC [14, 33].

Quality intrapartum care was defined as receiving all three of the following interventions during intrapartum care: a healthcare facility birth; skilled personnel-assisted birth; and the newborn put to the breast within one hour of birth [33, 34]. In the context of a shortage of skilled personnel in low-resource settings, the over-medicalization of normal childbirth can overburden front-line health workers, resulting in poor birth outcomes. Therefore, it is crucial that intrapartum clinical interventions are implemented only when there is clear evidence that they can actually improve outcomes and minimize potential harms [35]. The non-clinical aspects of labour and childbirth care, including emotional support through labour companionship, effective communication, and respectful care, are essential components of the care experience that should complement any necessary clinical interventions to optimize the quality of care provided to the woman and her family [34].

Quality PNC was defined as the receipt of all six of the following interventions during PNC: postnatal check for the mother and/or newborn within two days of birth at home or healthcare facilities; cord examination for the newborn; temperature measurement for the newborn; counselling on danger signs of newborn health; counselling on breastfeeding; and healthcare provider's observation of breastfeeding [33, 36].

### Explanatory variables

The explanatory variables included in this study were selected based on their significant associations with the outcome variables of interest in the literature and their availability in the DHS dataset [20, 21, 27, 32, 37–39]. Our study's quality ANC analyses included six explanatory socio-demographic and maternal healthcare variables: woman's education level (i.e., no formal education, primary education, or secondary or above), household wealth index (i.e., wealthiest, wealthier, middle, poorer, or poorest: the DHS wealth index is a composite indicator which divides the households into five categories: poorest, poorer, middle, wealthier and the richest, and were derived using principal component analysis based on information from housing characteristics and ownership of durable household goods) [40], area of residence (i.e., urban or rural: urban areas include all capitals of administrative regions, zones, and districts; rural areas are all areas that are not urban.), as well as cities (i.e., Addis Ababa and Dire Dawa) and administrative regions (i.e., Tigray, Afar, Amhara, Oromia, Somali, Benishangul-Gumuz, South Ethiopia, Gambela, and Harari), whether the woman received first ANC during the first trimester, number of ANC visits received (1–3, or ≥ 4), and type of healthcare facility where ANC was attended (government health post, public or private hospital, government health centre, or private clinic/NGO healthcare facility). The study's quality intrapartum care analyses included six explanatory socio-demographic and maternal healthcare variables: woman's education level, mother's age at birth (i.e., 10–19, 20–34 years, or 35–49 years), household wealth index, area of residence, whether the woman received four or more ANC visits during the pregnancy, and whether the woman received quality ANC during the pregnancy. Quality ANC was defined as having: blood pressure measurement, urine and blood tests, iron supplementation, informed of danger signs of pregnancy, and provision of nutritional counselling during the ANC visits for the last birth within five years preceding the survey. Our study's quality postnatal care analyses included eight explanatory socio-demographic and maternal healthcare variables: woman's education level, household wealth index, area of residence, mother's age at birth, preceding birth interval (i.e., < 24 months, or ≥ 24 months), whether the woman received first ANC during the first trimester, whether the woman received quality ANC during the pregnancy, and whether the woman received skilled-personnel-assisted birth during intrapartum care. Skilled personnel includes doctor, nurse, midwife, health officer, and health extension worker [29].

### Statistical analyses

The multilevel mixed-effects logistic regression (melogit) fits mixed-effects models for binary and binomial responses. The conditional distribution of the response given the random effects is assumed to be Bernoulli, with success probability determined by the logistic cumulative distribution function:

$$\mathrm{melogit\,y\,x }[\mathrm{iw}=\mathrm{sw}] ||\mathrm{ psu}:||\mathrm{ ssu}:,\mathrm{ or}$$

**Fig. 1:**
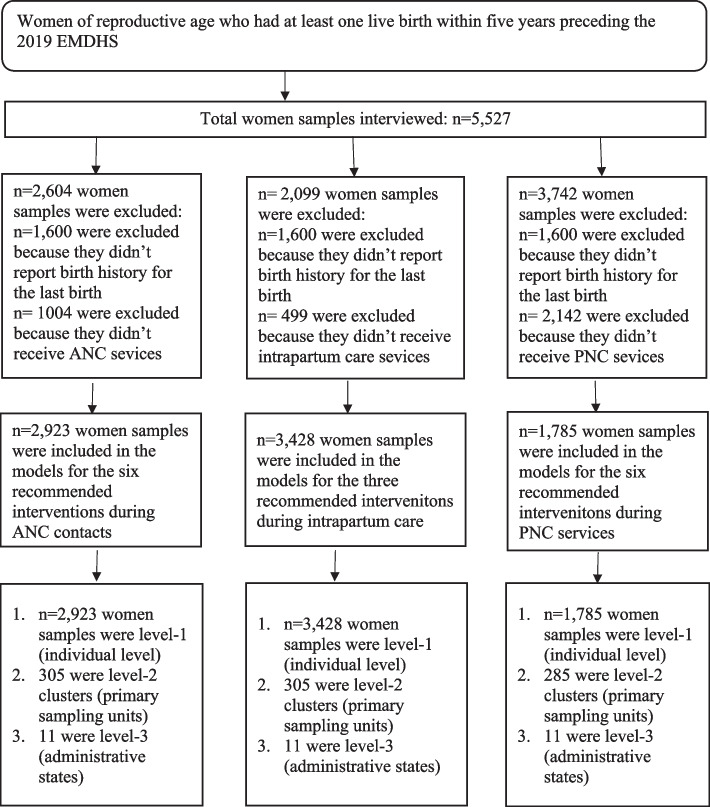
Schematic presentation showing included samples for quality ANC, intrapartum, and PNC analyses, Ethiopia MDHS 2019

The melogit models were used to determine the association between study characteristics at different levels and measures of quality of maternity care services (quality ANC, intrapartum care and PNC services). The 2019 EMDHS sample was stratified and selected in multiple stages generating hierarchically nested data: individual women were nested within EAs (clusters), and EAs (clusters) were nested within the administrative regions. Hence, the likelihood of women seeking quality ANC, intrapartum care or PNC services is likely to be correlated to the EA (cluster) members and residents of an administrative region. The utilization patterns of quality ANC, intrapartum care, or PNC services are influenced by the characteristics of different levels (individual women, EAs (clusters), and administrative regions) [41–44]. Assumptions of independence within a cluster (group) and equal variance across clusters (groups) are invalid for nested data [41]. The melogit modelling techniques allow us to simultaneously assess the variation of an outcome variable at several levels (individual women, EAs, and administrative regions). Aggregating or disaggregating variables to a single common level using the standard binary logistic regression model leads to bias (loss of power or Type I error) [41–45]. The melogit modelings allow us to consider the individual women level, EA (cluster) level, and administrative region level variables in the same analysis rather than having to choose one of the three [41–44]. Hence, the melogit modelling is the appropriate analytical technique for such data [46]. Accordingly, three-level models were specified. The level-one variables refer to individual women/household-level determinants, including socio-demographic and economic characteristics. At level two, we adjusted for clustering (EA) and included a primary sampling unit for analyses. While at level three, we adjusted for an administrative region where two cities and nine administrative regions were included in the analyses (Fig. 1). The melogit analyses were started from the intercept-only (null) models to test the null hypotheses that there were no variations in the women's utilisation of quality ANC, intrapartum care, or PNC services between administrative regions or between primary sampling units (EAs) in Ethiopia. For each outcome variable, we estimated two models: the null model (intercept-only model), an empty model with no independent variables, and a full model containing the individual women, EA (cluster), and administrative region level predictors. The null model was used to estimate the overall log of odds of each outcome variable across all individual, level-two, and level-three variables. It was used to check the significance of the association between each outcome variable and all individual women, EA (cluster) and administrative region level variables (fixed effects), and to estimate the intra-class correlation coefficient (Rho). Intra-class correlation coefficient (ICC) is the extent of the between classes variation influencing individual-level outcomes (random effects) [41, 47]. The ICC informs the researcher whether the variation in the scores is primarily within or between groups [41, 47]. Using the statistical software program: Stata/SE 16.1, all analyses employed sampling weights and were adjusted for sampling design (i.e., clustering and stratification). Statistical significance was set at *p* < 0.05.

Assume the binary quality maternal healthcare service response (quality ANC, intrapartum care or PNC services utilisation), ${\varvec{Y}}{\varvec{i}}{\varvec{j}}{\varvec{z}}$ depends on individual-level explanatory variable ${\varvec{X}}{\varvec{i}}{\varvec{j}}{\varvec{z}}$, EA/cluster-level explanatory variable ${\varvec{K}}{\varvec{j}}{\varvec{z}}$*,* and administrative region-level explanatory variable **R**_**z**_, then the logit function for the melogit modelling (log odds of outcome) will be:

$${\varvec{\eta}}{\varvec{i}}{\varvec{j}}{\varvec{z}}\boldsymbol{ }={\varvec{\beta}}0\boldsymbol{ }+\boldsymbol{ }{\varvec{\beta}}1\boldsymbol{^{\prime}}{\varvec{X}}{\varvec{i}}{\varvec{j}}{\varvec{z}}\boldsymbol{ }+\boldsymbol{ }{\varvec{\beta}}2\boldsymbol{^{\prime}}{\varvec{X}}{\varvec{K}}\boldsymbol{ }({\varvec{j}}{\varvec{z}})\boldsymbol{ }+\boldsymbol{ }{\varvec{\beta}}3\boldsymbol{^{\prime}}{\varvec{X}}{\varvec{R}}\boldsymbol{ }({\varvec{z}})\boldsymbol{ }+\boldsymbol{ }{\varvec{\varepsilon}}(2){\varvec{j}}({\varvec{z}})\boldsymbol{ }+\boldsymbol{ }{\varvec{\varepsilon}}(3){\varvec{z}}$$

where ${\varvec{\eta}}{\varvec{i}}{\varvec{j}}{\varvec{z}}=\boldsymbol{ }{\varvec{l}}{\varvec{n}}\boldsymbol{ }\{{\varvec{\pi}}{\varvec{i}}{\varvec{j}}{\varvec{z}}\boldsymbol{ }/\boldsymbol{ }1-\boldsymbol{ }{\varvec{\pi}}{\varvec{i}}{\varvec{j}}{\varvec{z}}\boldsymbol{ }\},\boldsymbol{ }{\varvec{\pi}}{\varvec{i}}{\varvec{j}}{\varvec{z}}$ denotes the probability that the ${\varvec{i}}{\varvec{t}}{\varvec{h}}$ mother in the ${\varvec{j}}{\varvec{t}}{\varvec{h}}$ 2^nd^ level cluster and the **z**^**th**^ 3^rd^ level cluster uses a quality maternal healthcare service (quality ANC, intrapartum care or PNC) ${\varvec{\pi}}{\varvec{i}}{\varvec{j}}{\varvec{z}}\boldsymbol{ }= P(Yijz =1), {\varvec{X}}{\varvec{i}}{\varvec{j}}{\varvec{z}}$ denotes the vector of the individual level variables, ${\varvec{X}}{\varvec{K}}{\varvec{j}}{\varvec{z}}$ denotes the vector of the second level variables, and ${\varvec{X}}{\varvec{R}}{\varvec{z}}$ denotes the vector of 3rd level predictor variables. In addition, ${\varvec{\beta}}1$ denotes the vector of regression parameters for the individual level variables, ${\varvec{\beta}}2$ denotes the vector of regression parameters for second level variables, and ${\varvec{\beta}}3$ denotes the vector of regression parameters for the 3rd level variables. Furthermore, ${\varvec{\varepsilon}}(2){\varvec{j}}({\varvec{z}})$ denotes the random effect for the ${\varvec{j}}{\varvec{t}}{\varvec{h}}$ second level cluster in the ${\varvec{z}}{\varvec{t}}{\varvec{h}}$ level cluster, and **ε**^**(3)**^_**z**_ denotes the random effect for the ${\varvec{z}}{\varvec{t}}{\varvec{h}}$ 3rd level cluster [48].

## Results

A total of 5,527 reproductive-age women (15–49 years) who gave birth within five years preceding the survey were included in the analyses. The majority of these women (75%) resided in rural areas, had no formal education (54%), were from poor households (46%), and gave their last birth at 20–34 years of age (74%).

### Quality ANC

**Fig. 2:**
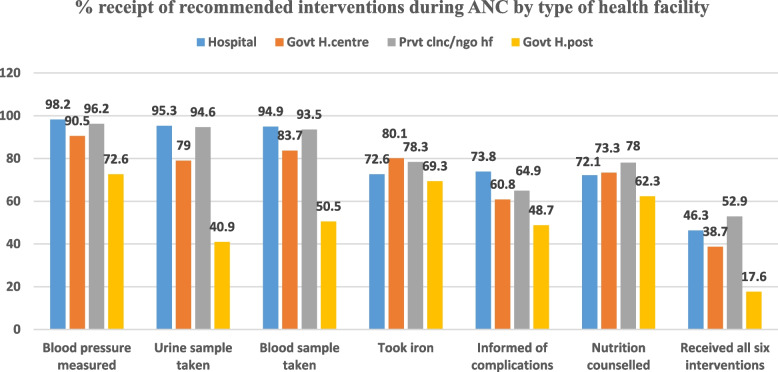
The percentage of women who received the recommended interventions during ANC visits by the type of health facility visited, Ethiopia MDHS 2019

In Ethiopia, 74% of women aged 15–49 years received at least one ANC visit for their last birth within five years preceding the 2019 Ethiopia MDHS, but only 43% of the women received four or more ANC visits for the last birth. In addition, only 28% of the women commenced their ANC contact during the first trimester. Of the women who attended at least one ANC visit for the last birth within five years preceding the 2019 Ethiopia MDHS, 88% reported blood pressure measurement, 79% reported blood tests to screen anaemia or infections, 77% received iron supplementation, 74% reported urine examination, 71% received nutritional counselling, and 60% were informed of pregnancy-related complications. All six of the recommended ANC interventions were received by 36% of women during their ANC visits (Table 1). The majority (53%) of women who had their ANC visits for their last birth at private clinics/NGOs received all the six recommended interventions during ANC, while only 18% of those who had their ANC visits for their last birth at government health posts received all the six recommended interventions during ANC (Fig. 2). In the Ethiopian Somali region, only 15% of the women who received ANC services for the last birth received all the six recommended interventions during ANC. In contrast, in the capital, Addis Ababa, 60% of the women who received ANC services for the last birth received all the six recommended interventions during ANC (Table 2).

**Table 1:** Quality antenatal care during pregnancy of the most recent live birth within five years preceding the survey, Ethiopia MDHS 2019 (*n* = 2,923)

ANC interventions	Frequency	Percent (%)	(95% CI.)
Blood pressure measured	2,575	88.1	(86.9, 89.3)
Urine sample taken	2,160	73.9	(72.3, 75.5)
Blood sample taken	2,305	78.9	(77.4, 80.4)
Informed of pregnancy complications	1,751	59.9	(58.1, 61.7)
Iron supplemented	2,249	77.0	(75.5, 78.5)
Nutritional counselling provided	2,077	71.0	(69.4, 72.6)
Received all six recommended interventions during ANC	1,048	35.9	(34.2, 37.6)

**Table 2:** Women's use of quality antenatal care services during pregnancy of the last birth within five years preceding the survey by administrative regions, Ethiopia MDHS 2019 (*n* = 2,923)

Regions	Frequency	Percent (%)	(95% CI.)
Tigray	130	47.9	(46.1, 49.7)
Afar	12	35.8	(34.1, 37.5)
Amhara	314	44.0	(42.2, 45.8)
Oromia	305	28.3	(26.7, 29.9)
Somali	10	14.5	(13.2, 15.8)
Benishangul G	12	29.7	(28.0, 31.4)
Southern Ethiopia	177	31.6	(29.9, 33.3)
Gambella	6	36.8	(35.1, 38.5)
Harari	3	37.1	(35.3, 38.9)
Addis Ababa	73	59.6	(57.8, 61.4)
Dire Dawa	7	40.8	(39.0, 42.6)

Of the women who received ANC for their most recent live births within five years preceding the survey, 37% commenced ANC during the first trimester, 58% received at least four ANC visits, while 3% attended their ANC in private clinics/NGO healthcare facilities and 66% in government health centres (Table 3).

**Table 3:** Determinants of receipt of all the six recommended components of ANC for the most recent live birth within five years preceding the survey, Ethiopia MDHS 2019 (*n* = 2,923)

Study variables	Total number of women (%)	Women who received all the six interventions during ANC (%)	Unadjusted Odds Ratio (95% CI.)	*p*-value	* Adjusted Odds Ratio (95% CI.)	*p*-value
**First trimester ANC**						
No	1,831 (62.6)	565 (30.9)	1.00		1.00	
Yes	1,092 (37.4)	483 (44.2)	2.66 (2.21, 3.19)	< 0.001	1.30 (1.07, 1.59)	0.009
**Number of ANC visits**						
1–3	1,225 (42.0)	304 (24.8)	1.00		1.00	
≥ 4	1,688 (58.0)	739 (43.8)	2.23 (1.85, 2.68)	< 0.001	1.88 (1.54, 2.29)	< 0.001
**Mother's level of education**						
No education	1,282 (43.9)	377 (29.4)	1.00		1.00	
Primary education (1–8)	1,153 (39.5)	416 (36.1)	1.82 (1.50, 2.21)	< 0.001	1.31 (1.06, 1.63)	0.012
Secondary or higher	487 (16.7)	255 (52.3)	3.51 (2.69, 4.59)	< 0.001	1.58 (1.18, 2.12)	0.002
**The place where the woman received ANC**						
Government H. post	559 (19.4)	98 (17.6)	1.00		1.00	
Hospital (public/private)	342 (11.9)	159 (46.3)	3.49 (2.37, 5.14)	< 0.001	2.19 (1.44, 3.32)	< 0.001
Government H. centre	1,902 (65.9)	736 (38.7)	2.58 (1.92, 3.46)	< 0.001	2.12 (1.57, 2.89)	< 0.001
Private clinic/NGO HF	82 (2.8)	43 (52.9)	5.52 (3.00, 10.30)	< 0.001	3.54 (1.87, 6.72)	< 0.001
**Household wealth quintile**						
Wealthiest	399 (13.7)	84 (20.9)	1.00		1.00	
Wealthier	587 (20.1)	160 (27.2)	0.37 (0.27, 0.51)	< 0.001	0.56 (0.39, 0.82)	0.002
Middle	589 (20.1)	206 (34.9)	0.34 (0.24, 0.47)	< 0.001	0.64 (0.43, 0.95)	0.028
Poorer	578 (19.8)	198 (34.3)	0.23 (0.16, 0.32)	< 0.001	0.47 (0.31, 0.71)	< 0.001
Poorest	770 (26.4)	401 (52.0)	0.12 (0.08, 0.17)	< 0.001	0.34 (0.21, 0.55)	< 0.001
**Mother's place of residence**						
Rural	2,052 (70.2)	664 (32.4)	1.00		1.00	
Urban	871 (29.8)	384 (44.1)	2.44 (1.56, 3.80)	<0.001	0.74 (0.46, 1.18)	0.201

^*^ Odds ratio adjusted for all study variables listed in the table

### Factors associated with the receipt of all the six recommended interventions during ANC

Mothers who had their first ANC contact during the first trimester were 30% more likely (AOR = 1.30; 95% CI = 1.07, 1.59) to receive all the six recommended interventions during ANC visits, compared to women who had no or late ANC contact. Mothers who had four or more ANC visits for the last birth were 88% (AOR = 1.88; 95% CI = 1.54, 2.29) more likely to receive all the six recommended interventions during ANC visits than women who had fewer ANC contacts. Receipt of all the six recommended interventions during ANC was 11 percentage points and 33 percentage points higher among mothers with primary education and secondary or higher education levels, respectively, compared with mothers with no education. With increasing levels of mother's education, the likelihood of receiving all the six recommended interventions during ANC significantly increased; mothers with secondary or higher education were 58% (AOR = 1.58; 95% CI = 1.18, 2.12) more likely to receive all six recommended interventions, compared to mothers who had no formal education. Mothers who received ANC for their last birth from government health posts were less likely to receive all the six recommended interventions; mothers who received their ANC at private clinics and/or NGOs, at hospitals, and at government health centres were 3.54 times (AOR = 3.54; 95% CI = 1.87,6.72), 2.19 times (AOR = 2.19; 95% CI = 1.44, 3.32) and 2.12 times (AOR = 2.12; 95% CI = 1.57, 2.87) more likely to receive the six recommended interventions during ANC than mothers who received ANC at government health posts, respectively. The receipt of all the six recommended interventions during ANC visits was 39 percentage points higher among the richest mothers compared with the poorest mothers. With decreasing indices of households' wealth index, the likelihood of receiving all six recommended interventions during ANC visits also significantly decreased; mothers from households with the poorest wealth indices were 66% (AOR = 0.34; 95% CI = 0.21, 0.55) less likely, compared to mothers from households with the richest wealth indices (Table 3).

### Quality intrapartum care

**Fig. 3:**
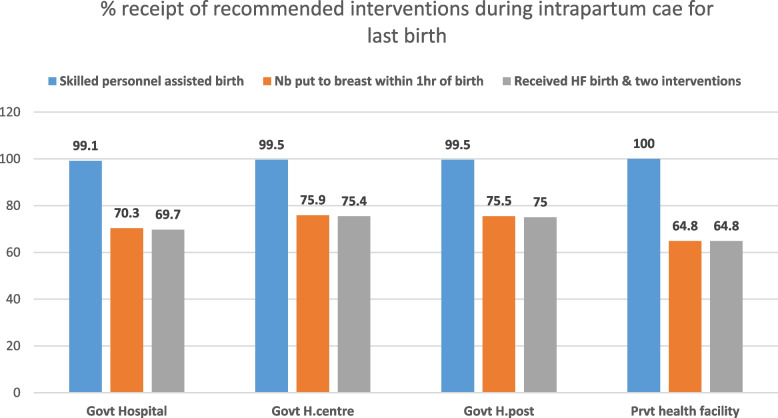
The percentage of mothers receiving the recommended interventions during intrapartum care by type of health facility, Ethiopia MDHS 2019

In Ethiopia, 87% of women aged 15–49 years received assistance during labour and delivery (intrapartum care) for the last birth within five years preceding the 2019 Ethiopia MDHS. However, of the women who received intrapartum care during labour and delivery for their most recent live births within five years preceding the 2019 Ethiopia MDHS, only 59% gave birth in healthcare facilities. In addition, of the women who received intrapartum care for the last birth within five years preceding the survey, only 62% had skilled personnel-assisted birth, and 72% had their newborn put to their breast within one hour of birth. Furthermore, of the women who received intrapartum care for the last birth within five years preceding the survey, only 43% received all the three recommended interventions (Table 4). The majority (75%) of women who had their intrapartum care for their last birth at government health centres received all the three recommended intrapartum care interventions, while only 65% of those who had their intrapartum care for the last birth at private healthcare facilities received all the three recommended intrapartum care interventions (Fig. 3). In the Ethiopian Somali region, only 17% of the women who received intrapartum care for the last birth received all the three recommended intrapartum care interventions. In contrast, in the capital, Addis Ababa, 67% of the women who received intrapartum care for the last birth received all the three recommended interventions during intrapartum care (Table 5).

**Table 4:** Three recommended interventions during intrapartum care for the most recent live birth within five years preceding the survey, Ethiopia MDHS 2019 (*n* = 3,428)

Intrapartum care interventions	Frequency	Percent	(95% CI.)
Health facility birth	2,018	58.9	(57.3, 60.5)
Skilled personnel assisted birth	2,120	61.8	(60.2, 63.4)
Newborn put to the breast within 1 h of birth	2,464	71.9	(70.4, 73.4)
Received three recommended interventions during the intrapartum care	1,485	43.3	(41.6, 45.0)

**Table 5:** Women's use of quality intrapartum care for the last birth within five years preceding the survey by administrative regions, Ethiopia MDHS 2019 (*n* = 3,428)

Regions	Frequency	Percent (%)	(95% CI.)
Tigray	162	58.0	(56.3, 59.7)
Afar	15	29.8	(28.3, 31.3)
Amhara	302	40.0	(38.4, 41.6)
Oromia	565	46.2	(44.5, 47.9)
Somali	37	17.4	(16.1, 18.7)
Benishangul G	25	66.4	(64.8, 68.0)
Southern Ethiopia	267	38.6	(37.0, 40.2)
Gambella	11	58.9	(57.3, 60.5)
Harari	6	53.8	(52.1, 55.5)
Addis Ababa	85	67.4	(65.8, 69.0)
Dire Dawa	10	50.1	(48.4, 51.8)

Of the women who received intrapartum care during labour and delivery for their most recent live births within five years preceding the survey, 47% had received at least four ANC visits, and 29% had received the six recommended interventions during ANC visits (Table 6).

**Table 6:** Determinants of receipt of all the three recommended components of intrapartum care for the most recent live birth within five years preceding survey, Ethiopia MDHS 2019 (*n* = 3,428)

Study variables	Total number of women (column %)	Women who received the three interventions during intrapartum care (%)	Unadjusted Odds Ratio (95% CI.)	*p*-value	^a^Adjusted Odds Ratio (95% CI.)	*p*-value
**Four or more ANC visits**						
No	1,828 (53.3)	562 (30.7)	1.00		1.00	
Yes	1,600 (46.7)	923 (57.7)	2.16 (1.81, 2.57)	<0.001	1.80 (1.50, 2.17)	<0.001
**Received quality ANC for the last birth**						
No	2,429 (70.8)	907 (37.3)	1.00		1.00	
Yes	1,000 (29.2)	578 (57.8)	1.64 (1.35, 1.98)	<0.001	1.22 (0.99, 1.49)	0.058
**Mother's level of education**						
No education	1,650 (48.1)	521 (31.6)	1.00		1.00	
Primary education (1–8)	1,287 (37.5)	638 (49.6)	1.88 (1.55, 2.27)	<0.001	1.57 (1.29, 1.93)	<0.001
Secondary or higher	492 (14.4)	325 (66.1)	3.12 (2.37, 4.10)	<0.001	2.00 (1.49, 2.67)	<0.001
**Mother's age at birth (years)**						
12–19	501 (14.6) 2,474	244 (48.8)	1.00		1.0	
20–34	(72.2)	1,085 (43.9)	0.75 (0.59, 0.95)	0.016	0.73 (0.57, 0.93)	0.012
35–49	453 (13.2)	156 (34.4)	0.52 (0.38, 0.71)	<0.001	0.63 (0.45, 0.88)	0.007
**Household wealth quintile**						
Wealthiest	788 (23.0)	526 (66.8)	1.00		1.00	
Wealthier	624 (18.2)	317 (50.9)	0.55 (0.40, 0.76)	<0.001	0.78 (0.55, 1.10)	0.159
Middle	677 (19.7)	235 (34.8)	0.28 (0.20, 0.40)	<0.001	0.45 (0.31, 0.66)	<0.001
Poorer	687 (20.1)	274 (39.9)	0.37 (0.26, 0.52)	<0.001	0.62 (0.42, 0.92)	0.017
Poorest	652 (19.0)	132 (20.2)	0.17 (0.11, 0.24)	<0.001	0.32 (0.21, 0.50)	< 0.001
**Mother's place of residence**						
Rural	2,462 (71.8)	934 (37.9)	1.00		1.00	
Urban	966 (28.2)	551 (57.0)	3.84 (2.39, 6.16)	<0.001	1.66 (1.04, 2.64)	0.034

^a^ Odds ratio adjusted for all study variables listed in the table

### Factors associated with the receipt of all the three recommended interventions during intrapartum care

Mothers who had four or more ANC visits for the last birth were 80% (AOR = 1.80; 95% CI = 1.50, 2.17) more likely to receive all the recommended three interventions during intrapartum care than mothers who had no or fewer ANC contacts during pregnancy. The likelihood of receiving all three interventions during intrapartum care increased with the mother's education; mothers with secondary or higher education levels were two times (AOR = 2.00; 95% CI = 1.49, 2.67) more likely than mothers who had no formal education. The likelihood of receiving all three recommended interventions during intrapartum care decreased with increasing maternal age; advanced-age mothers (35–49 years) were 37% less likely than teenage mothers (AOR = 0.63; 95% CI = 0.45, 0.88). With decreasing indices of households' wealth index, the likelihood of receiving all three interventions during intrapartum care also decreased; where mothers from households with the poorest wealth indices being 68% (AOR = 0.32; 95% CI = 0.21, 0.50) less likely, compared to mothers from households with the richest wealth indices. Mothers residing in an urban area were 66% (AOR = 1.66; 95% CI = 1.04, 2.64) more likely to receive all three interventions during intrapartum care than rural mothers (Table 6).

### Quality PNC

**Fig. 4:**
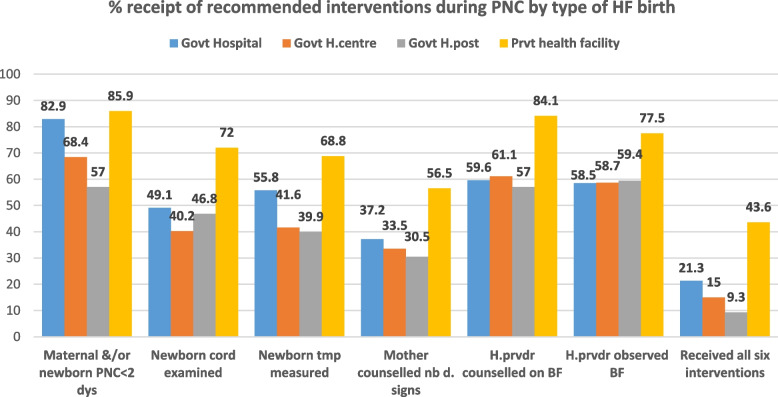
The percentage of mothers and/or their newborns who received the recommended interventions during PNC by type of health facility where the birth occurred, Ethiopia MDHS 2019

In Ethiopia, 45% of women aged 15–49 years and/or their newborns received PNC for the last birth within five years preceding the 2019 Ethiopia MDHS. However, only 33% of women aged 15–49 years and 32% of newborns, respectively, received PNC within two days of birth for the last birth within five years preceding the 2019 Ethiopia MDHS. Among mothers and/or their newborns who received PNC at home or in healthcare facilities for the most recent live births within five years preceding the 2019 Ethiopia MDHS, 87% received maternal and/or newborn PNC within two days of birth; a healthcare provider counselled the mother on breastfeeding in 63% of births; a healthcare provider observed the newborn breastfeeding in 59% of births; a healthcare provider measured the newborn's temperature in 51% of births; a healthcare provider examined the newborn's cord in 49% of births, and a healthcare provider counselled the mother on newborn danger signs in 39% of births. All these six recommended interventions were received by 21% of women and/or their newborns during postnatal care (Table 7). Among women who gave birth at private healthcare facilities, 44% received all six recommended PNC interventions. In contrast, among those who gave birth at government health posts, only 9% received all six recommended PNC interventions (Fig. 4). In Ethiopia's Harari region, only 11% of the women and/or their newborns who received PNC services for the last birth received all the six recommended PNC interventions. In contrast, in the capital, Addis Ababa, 47% of the women and/or their newborns who received PNC services for the last birth received all the six recommended PNC interventions (Table 8).

**Table 7:** Quality maternal and/or newborn PNC for the most recent live birth within five years preceding the survey, Ethiopia MDHS 2019 (*n* = 1,785)

PNC interventions	Frequency	Percent	(95% CI.)
Maternal and/or newborn PNC within two days of birth	1,559	87.3	(85.8, 88.8)
The health provider examined the newborn cord	877	49.2	(46.9, 51.5)
The health provider measured newborn temperature	902	50.6	(44.1, 48.7)
Health provider counselled mother on newborn's health danger signs	691	38.7	(36.4, 41.0)
Health provider counselled on breastfeeding	1,124	63.0	(60.8, 65.2)
The health provider observed breastfeeding	1,046	58.6	(56.3, 60.9)
Received all six recommended interventions during PNC	374	20.9	(19.0, 22.8)

**Table 8:** Women's use of quality PNC for the last birth within five years preceding the survey by administrative regions, Ethiopia MDHS 2019 (*n* = 1,785)

Regions	Frequency	Percent (%)	(95% CI.)
Tigray	58	27.5	(25.4, 29.6)
Afar	5	32.7	(30.5, 34.9)
Amhara	68	15.4	(13.7, 17.1)
Oromia	95	16.2	(14.5, 17.9)
Somali	11	25.0	(23.0, 27.0)
Benishangul G	9	38.0	(35.7, 40.3)
Southern Ethiopia	65	20.8	(18.9, 22.7)
Gambella	6	43.2	(40.9, 45.5)
Harari	1	10.7	(9.3, 12.1)
Addis Ababa	54	46.8	(44.5, 49.1)
Dire Dawa	2	12.6	(11.1, 14.1)

Among mothers with the most recent live births within five years preceding the survey and who received maternal and/or newborn PNC services, 90% had skilled personnel-assisted birth; 87% had a preceding birth interval of 24 or more months; 41% had commenced ANC during the first trimester, and 40% had received the six recommended interventions during ANC visits (Table 9).

### Factors associated with the receipt of all the six recommended interventions during maternal and/or newborn PNC services

Mothers who had commenced ANC contact during the first trimester were 2.2 times (AOR = 2.20; AOR = 1.56, 3.11) more likely to receive all six interventions during PNC services, compared to women who had had no or late ANC contact. Mothers who had received the six recommended interventions during ANC for pregnancy of last birth were 2.52 times (AOR = 2.52; 95% CI = 1.79, 3.55) more likely to receive all the six recommended interventions during maternal and/or newborn PNC than mothers who had received less or no interventions during their ANC. Mothers who received skilled personnel-assisted birth were 5.55 times (AOR = 5.55; 95% CI = 2.54, 12.17) more likely to receive all six interventions during the PNC than mothers who did not receive skilled personnel assistance during labour and delivery. Mothers aged 20–34 years and the elderly (35–49 years) were 5.44 times (AOR = 5.44; 95% CI = 1.90, 15.56) and 4.72 times (AOR = 4.72; 95% CI = 1.56, 14.27) more likely to receive all the six recommended interventions during PNC, respectively than teenage mothers. Mothers with 24 or more months of preceding birth intervals were 59% (AOR = 0.41; 95% CI = 0.26, 0.66) less likely to receive all six interventions during maternal and/or newborn PNC services than mothers with shorter preceding birth intervals (Table 9).

**Table 9:** Determinants of receipt of all the six recommended interventions during maternal and/or newborn PNC for the most recent live birth within five years preceding survey, Ethiopia MDHS 2019 (*n* = 1,785)

Study variables	Total number of women (column %)	Women who received all the six interventions during PNC (%)	Unadjusted Odds Ratio (95% CI.)	*p*-value	*Adjusted Odds Ratio (95% CI.)	*p*-value
**First trimester ANC**						
No	1,057 (59.2)	181 (17.1)	1.00		1.00	
Yes	728 (40.8)	193 (26.5)	1.72 (1.32, 2.26)	<0.001	2.20 (1.56, 3.11)	<0.001
**Received quality ANC for last birth**						
No	1,065 (59.7)	151 (14.2)	1.00		1.00	
Yes	720 (40.3)	223 (30.9)	2.59 (1.98, 3.40)	<0.001	2.52 (1.79, 3.55)	<0.001
**Skilled personnel assisted birth**						
No	184 (10.3)	12 (6.6)	1.00		1.00	
Yes	1,601 (89.7)	362 (22.6)	4.34 (2.26, 8.35)	<0.001	5.55 (2.54, 12.17)	<0.001
**Mother's age at birth (years)**						
12–19	262 (14.7)	24 (9.3)	1.00		1.00	
20–34	1,302 (72.9)	302 (23.2)	2.85 (1.78, 4.55)	<0.001	5.44 (1.90, 15.56)	0.002
35–49	221 (12.4)	48 (21.6)	2.44 (1.38, 4.32)	0.002	4.72 (1.56, 14.27)	0.006
**Mother's level of education**						
No education	652 (36.5)	133 (20.5)	1.00		1.00	
Primary education (1–8)	738 (41.4)	120 (16.3)	0.80 (0.59, 1.12)	0.179	0.74 (0.50, 1.11)	0.145
Secondary or higher	395 (22.1)	121 (30.5)	1.54 (1.07, 2.22)	0.019	1.17 (0.69, 1.98)	0.568
**Preceding birth interval**						
< 24 months	169 (13.4)	53 (31.6)	1.00		1.00	
≥ 24 months	1,096 (86.7)	237 (21.6)	0.54 (0.35, 0.83)	0.005	0.41 (0.26, 0.66)	<0.001
**Household wealth quintile**						
Wealthiest	611 (34.2)	161 (26.4)	1.00		1.00	
Wealthier	373 (20.9)	64 (17.1)	0.85 (0.52, 1.37)	0.501	1.47 (0.75, 2.86)	0.263
Middle	325 (18.2)	62 (18.9)	0.89 (0.54, 1.47)	0.652	1.50 (0.74, 3.04)	0.255
Poorer	294 (16.5)	52 (17.8)	0.79 (0.47, 1.32)	0.373	0.95 (0.46, 1.97)	0.894
Poorest	182 (10.2)	35 (19.2)	0.82 (0.46, 1.46)	0.497	1.30 (0.57, 2.95)	0.530
**Mother's place of residence**						
Rural	1,126 (63.1)	214 (19.0)	1.00		1.00	
Urban	659 (36.9)	159 (24.2)	1.05 (0.62, 1.79)	0.855	0.87 (0.41, 1.82)	0.708

^*^Odds ratio adjusted for all study variables listed in the table

## Discussion

Our study revealed that among mothers who accessed maternal healthcare for their most recent live birth in Ethiopia, only 36% received all six recommended interventions during their ANC. Forty-six percent received all the three recommended interventions during intrapartum care, and 21% received all six recommended interventions during the PNC.

### Quality ANC

Our study showed that commencing ANC early (during the first trimester) and having four or more ANC contacts significantly improved the likelihood of receiving all the six recommended interventions during ANC. Antenatal care is the routine care of pregnant women provided between conception and the onset of labour, and is an opportunity to provide care to prevent and manage existing and potential causes of maternal and newborn morbidity and mortality. The timing of the first ANC contact is paramount for ensuring optimal health outcomes for a woman and her child, and it is recommended that the first ANC contact takes place within the first trimester (i.e., gestational age of < 12 weeks) [14, 49]. The early and more frequently a mother seeks routine care during pregnancy, the more she receives recommended interventions during pregnancy intended to improve maternal and newborn health outcomes. The critical interventions during pregnancy: dietary interventions (counselling about healthy eating, nutrition education and screening, and keeping physically active during pregnancy); iron and folic acid supplementation; calcium supplementation (for those with low dietary calcium intake); vitamin A supplementation (in vitamin A deficiency endemic areas); blood testing to screen anaemia, gestational diabetes mellitus, HIV/AIDS and syphilis; urine testing to screen asymptomatic bacteriuria; early clinical screening (high caffeine intake, tobacco, drug use, intimate partner violence, tuberculosis screening in high prevalence areas, and ultrasound scan) are all recommended to be commenced or provided during the first visit and in the first trimester [14]. Our finding concurs with a study based on the secondary data analysis of the latest demographic and health surveys (2013–2018) from nine East African countries, including Ethiopia, by Bobo et al. (2021), which showed that women who commenced ANC during the first trimester were 29% more likely to receive six recommended interventions during ANC than those who commenced ANC lately, while women who had received four or more ANC visits were 37% more likely to receive the six recommended interventions during ANC than those who received less ANC visits [32]. Secondary data analysis of Rwanda's 2020 DHS by Sserwanja et al. [2022] also revealed that women who commenced ANC during the first trimester were 45% more likely to receive six recommended interventions during ANC than those who commenced ANC lately, while women who had received four or more ANC visits were 52% more likely to receive the six recommended interventions during ANC than those who received less ANC visits [50].

Significant and positive associations also exist between increasing the mother's education level or household's wealth quintile and receiving the six recommended interventions during ANC. The knowledge and skills attained through education positively affect a person's cognitive functioning, make one more receptive to health education messages, or enable one to communicate with and access appropriate health services. It captures the long-term influences of both early life circumstances on adult health and the influence of adult resources (e.g., through employment status) on health [51]. Household income is a proximate indicator of access to scarce material resources or a standard of living that allows a mother to directly access health services, which may improve health [51]. In the study by Bobo et al. (2021), secondary or higher maternal education level and highest household wealth index improved the likelihood of a mother receiving all the six recommended interventions during ANC visits by 28%, and 26% compared to no education, and poorest household wealth index, respectively [32]. Our findings are also similar to another population-based study using the 2011 Nepalese demographic and health survey by Joshi et al. [2014], which found that women with higher levels of education and those from higher wealth index households had higher odds of receiving all the recommended ANC components [30].

In Ethiopia, government health posts appear to be providing poor-quality ANC. This might be due to the lack of access to adequate equipment and supplies, lack of readiness, and lack of required expertise and skills among health providers in the health posts at village levels. These healthcare facilities are nearest to communities in need. The assessment of a healthcare facility's readiness and their health providers' knowledge of maternal and immediate newborn care to perform specific functions may assist in identifying those facilities in need of strengthening. A systematic review by Negero et al. (2021) revealed that in primary care settings in LLMICs, community-based or onsite health workforce interventions involving both skilled and lay personnel were more effective in improving Sexual, reproductive, maternal, and newborn health (SRMNH) care quality along the continuum than those involving either skilled or lay personnel alone [52]. These workforce interventions include education and training, policy, management and leadership strategies, partnerships, and results-based financing initiatives. The regular deployment of experienced personnel, equipment, and supplies from higher levels of care (health centres and hospitals) to the nearby health posts to reach out to less advantaged mothers and sharing skills and expertise is critical. A study in a rural district of Nepal showed that facility readiness to provide quality maternal and newborn care in rural districts was low, but changes including regular monitoring, improving staffing and supply chains, supportive supervision, and refresher training initiatives improved maternal and newborn healthcare quality [53]. In our study, private healthcare facilities had higher odds of providing quality ANC and PNC. This corroborates with two studies conducted in Addis Ababa, Ethiopia, and Southern Ethiopia, where clients at private healthcare facilities were two times more likely to receive all the recommended ANC components than those of public healthcare facilities [20, 21]. The limited availability of equipment, medications, and trained staff due to underfunding and poor management, the redirection of highly skilled staff to private healthcare facilities, and the lack of timeliness and hospitality pose a threat to optimal care at public healthcare facilities [54].

### Quality intrapartum care

In our study, frequent ANC visits (four or more ANC) improved the likelihood of receiving all the three recommended components of intrapartum care. This might be because the more frequently a mother encounters the healthcare system during pregnancy, the better her healthcare-seeking behaviour, birth preparedness, and complication readiness planning will be. This is also likely to lead to an increased demand for quality intrapartum care. A study based on the Bangladesh DHS 2014 data similarly showed a significant and positive association between having four or more ANC visits during pregnancy and receiving skilled personnel-assisted birth and healthcare facility birth [55]. Our study also shows significant and positive associations between higher women's education level, higher household wealth index and urban residence, and receipt of all the three recommended components of intrapartum care. Over 80% of Ethiopians dwell in rural areas, and home delivery is disproportionately higher among rural women [56, 57]. A review based on primary studies in Ethiopia by Kebede et al. [2016] similarly showed significant and positive associations between urban residence and primary and above-level women's education, and healthcare facility delivery service utilization [58]. Teenage mothers had higher odds of receiving all the three recommended components of intrapartum care than mothers aged 20–49 years. This may be because healthcare providers were more vigilant during the birth of a teenage's baby since teenage pregnancy is significantly related to adverse maternal and/or perinatal outcomes, including eclampsia, puerperal endometritis, systemic infections, preterm delivery, small for gestational age birth, stillbirth, and neonatal death. Preventing these poor health outcomes through increased use of skilled ANC, intrapartum, and PNC services for teenage mothers is recommended [59–61]. However, this finding contradicts a study from India where women aged 25–29, 30–34, and 35–39 years of age at birth, respectively, were 21% (AOR = 1.21; 95% CI = 1.00, 1.47), 71% (AOR = 1.71; 95%CI = 1.36, 2.15), and 51% (AOR = 1.51; 95%CI = 1.12, 2.03) more likely to receive quality intrapartum care services compared to teenage mothers [62].

### Quality PNC

Our study also revealed that commencing ANC contacts early (during the first trimester), receiving all the six recommended interventions during ANC visits and skilled personnel-assisted birth improved the likelihood of receiving all six interventions during PNC. Commencing ANC early, receiving the recommended interventions during ANC visits, and skilled personnel assistance at birth imply that the mother is more likely to be informed about complications that may occur after delivery and thus recognize the importance of seeking optimal care during postnatal care. Fekadu et al. (2019) showed that Ethiopian mothers who had four or more ANC contacts and received more content of care during ANC visits had higher odds of receiving PNC services [38]. Further, teenage mothers were disproportionately less likely to receive the recommended interventions during PNC than mothers aged 20–49 years. The significantly lower quality of care during PNC services for teenage mothers might be due to the providers' lack of knowledge about vulnerable groups of women and their associated judgmental attitudes, particularly towards unmarried teenagers. A teenage mother's emotional or intellectual immaturity, low education, or inexperience could also hinder her from seeking optimal care after birth. Therefore, it is necessary to provide teenage-friendly services to teenage mothers and their newborns after birth [63, 64]. Teenage mothers have until recently been overlooked in global health and social policy [65]. A study based on the secondary data analysis of a nationally representative survey from India, however, revealed that compared to teenage mothers, mothers aged 20–24, 25–29, 30–34, 35–39, and 40–49 years of age had no statistically significant association with the mother's use of quality PNC services [62].

Our study has revealed that there is much opportunity for improving the quality of antenatal, intrapartum, and PNC services in Ethiopia. Unfortunately, Ethiopia is presently facing significant challenges to healthcare delivery brought on by the COVID-19 pandemic and the conflict in northern Ethiopia. Quality maternal healthcare services that respond to the local need and meet emerging challenges, including nomadic-pastoralists, Covid-19, and conflicts, are critical. Mobile clinics, hospitals and mobile health (mHealth) digital solution services should be available near needy communities. Universal coverage of maternal healthcare quality should be promoted, including for the most vulnerable: poor, uneducated, rural women, and teenage mothers. The resilience and strength of the healthcare system in Ethiopia should be increased by optimizing the health workforce, equipment and supplies, and improving lower-level healthcare facility capability. The use of digital clinical decision support tools by health providers, guaranteeing sustainable finances for maternal and perinatal healthcare, and accelerating progress in maternal healthcare quality through evidence, advocacy, and accountability are needed [23, 24, 66–69]. In addition to evaluating the receipt of the recommended interventions during care services, assessing mothers' experiences and perceptions of the care services is also important and is likely to have a major effect on satisfaction and uptake of services [70]. Furthermore, it would be critical to strengthening health extension workers' preventive and health promotion services to encourage mothers to demand the recommended interventions during pregnancy, intrapartum, and postnatal care services. Currently, health extension workers in Ethiopia are no longer allowed to perfume delivery care services for mothers. They are rather expected to provide good-quality ANC services and postnatal home visits [71].

Our study was based on the latest (2019) nationally representative Ethiopia DHS data and employed multilevel modelling techniques for simultaneously identifying the potential predictors of the use of quality maternity care services at different levels. However, this study also had several limitations. We used the recommended components of maternal healthcare services mentioned in the WHO's guidelines to measure the quality of each maternal healthcare, which may not equate with the experience of care by the mother, i.e., client satisfaction. The mother reported the responses to the survey questionnaire concerning her most recent live birth within five years preceding the survey, with potential recall bias and lack of ability to identify between the different care providers (e.g., doctors, nurses). Due to the lack of data availability, some recommended components of each care service, including ANC contact schedules, ultrasound scan during the first 24 weeks of gestation, the experience of care (respectful care), effective communication, a companion of choice during labour and delivery, and delayed umbilical cord clamping were missing. The study also shares the limitations of a cross-sectional study design, which makes it difficult to demonstrate cause-and-effect relationships.

## Conclusions

This study shows that the recommended healthcare interventions women and/or their newborns receive during antenatal, intrapartum, or PNC services in Ethiopia were inadequate compared to the WHO's recommended intervention guidelines. Policy-makers, managers, and healthcare providers in Ethiopia should increase the quality of care provided to mothers and/or their newborns during antenatal, intrapartum, and PNC services as per the WHO's guidelines. In addition to strengthening health systems, community-based initiatives such as women's groups to raise awareness of the importance of early and frequent ANC contacts and linking pregnant mothers to the healthcare system are needed. There is a need to promote education for girls and women and enable women's economic empowerment. We also recommend that the DHS program incorporate pertinent questions to assess and promote other critical interventions, including early ultrasound scan during the first 24 months of pregnancy, delayed umbilical cord clamping, and care experience, including respectful care during care services.

## Data Availability

This study was based on analyses of an existing dataset in the DHS repository that is freely available online with permission to use and with all identifier information removed (https://dhsprogram.com/data/).

## Abbreviations

ANC: Antenatal care
PNC: Postnatal care
SRMNH: Sexual, reproductive, maternal, and newborn health
WHO: World Health Organization
